# *Oreocharisodontopetala*, a new species of Gesneriaceae from Guizhou, China

**DOI:** 10.3897/phytokeys.124.34609

**Published:** 2019-06-06

**Authors:** Qiong Fu, Ying Xia, Ying Guo, Rong Huang, Ying-Qiang Wang

**Affiliations:** 1 Guangdong Provincial Key Laboratory of Biotechnology for Plant Development, School of Life Sciences, South China Normal University, Guangzhou 510631, China; 2 Guangzhou Key Laboratory of Subtropical Biodiversity and Biomonitoring, School of Life Sciences, South China Normal University, Guangzhou 510631, China; 3 Panzhou Bureau of Agriculture and Rural Affairs, Panzhou, Guizhou 553537, China; 4 Liupanshui Niangniangshan National Wetland Park Administration Office, Panzhou, Guizhou 553522, China

**Keywords:** *
Briggsia
*, endemism, leaf epidermis, morphology, new taxon, taxonomy

## Abstract

A new species, *Oreocharisodontopetala* Q.Fu & Y.Q.Wang from Guizhou Province in southwest China, is described and illustrated, based on morphological comparison with existing species. It is morphologically most similar to *O.elegantissima*, but can be easily distinguished by its adaxially bullate leaf blade, abaxially conspicuous reticulate veinlets, brown-purple peduncles, triangular adaxial corolla lobes and abaxial corolla lobe margins bearing 4–10 long teeth, glabrous style and shorter stamens with confluent thecae at the apex, as well as leaf epidermal characters.

## Introduction

The genus *Briggsia* was established by Craib (1919a) and had at one point > 20 species (Wang et al. 1990, 1998; Li and Wang 2004; Wu et al. 2012; Wen et al. 2015). *Briggsia*, in its original definition, was described as having species with a large distinctly bilabiate ventricose corolla, four fertile stamens with the anthers cohered in pairs and gradually inarching filaments (Craib 1919b). However, the genus underwent various taxonomic changes and the rosette-forming species were recently subsumed in the enlarged genus *Oreocharis* that currently includes over 120 species (Möller et al. 2011, 2014, 2016, 2018). It is distributed in China, Thailand, Vietnam, Myanmar, Bhutan, NE India and Japan.

During our fieldwork on floral biology of *Oreocharis* in August 2018, we found an unrecognised species of *Oreocharis* resembling species of the former *Briggsia* on Wumeng grassland, Wumeng Town, Panzhou City, Guizhou Province, Southwest China. After carefully comparisons of diagnostic characters of *Oreocharis* specimens and consulting the relevant literature, we found it was most similar to *O.elegantissima* (H. Lév. & Vaniot) Mich.Möller & W.H.Chen (previously *Briggsiaelegantissima*), but was evidently different in leaf and flower morphology. Here, we describe and illustrate the unknown species as a new species of *Oreocharis*.

## Methods

In the flowering season 2018, comparative studies on the morphology and floral ecology between the new species and *Oreochariselegantissima* were carried out from two different localities. The new species grows on limestone rocks of a hill forest in Wumeng grassland, Wumeng Town, Panzhou City, Guizhou Province, southwest China (26°4.33'N, 104°37.34'E, alt. ca. 2100 m) and the studied population of *O.elegantissima* grows on limestone rocks in a subtropical moist forest in Heibai Village, Yuni Town, Panzhou City, Guizhou Province, southwest China (25°59.50'N, 104°51.27'E, alt. ca. 1760 m). Morphological observations and measurements were carried out on living plants, dried specimens and preserved materials under stereomicroscopes and morphological characters were described, following the terminology presented by Wang et al. (1998). Fresh experimental materials (including leaves, flowers and fruits) were obtained from the fields (at least 10 leaves and 10 flowers from 10 plants) and the micromorphological characters were further investigated with a transmission light microscope (Zeiss Axio Imager A1, Göttingen, Germany) and a scanning electron microscope (Jeol JSM-6360, Akishima, Japan). The materials for the LM study were boiled in water and then epidermal tissue was obtained from the leaves by tearing. The materials for SEM observations were macerated in 4% glutaric dialdehyde solution for about 24 hours, dehydrated in a gradient alcohol series and then critical point dried with Lecia EM CPD 300 (Vienna, Austria). Subsequently, samples were directly mounted on stubs and sputter-coated with gold-palladium. The terminology of micromorphological characters followed Dilcher (1974).

## Taxonomy

### Oreocharis odontopetala

Taxon classificationPlantaePasseriformesParamythiidae

Q.Fu & Y.Q.Wang sp. nov.

urn:lsid:ipni.org:names:60478958-2

Figures 1 , 2

#### Diagnosis.

*Oreocharisodontopetala* is most similar to *O.elegantissima*, having a similar shape of leaf blade, lanceolate sepals and bracts, stellate ring-like disc, pistil and fruits. *Oreocharisodontopetala* differs from *O.elegantissima* by its adaxially bullate leaf blade (vs. not bullate), with abaxially reticulate veinlets conspicuous (vs. veinlets inconspicuous); peduncles brown-purple (vs. green); adaxial corolla lobes triangular (vs. oblong) and abaxial corolla lobe margins with 4–10 long teeth (vs. margin nearly entire); style glabrous (vs. glandular pubescent) and stamens shorter (adaxial 0.5–1.4 vs. 2.0–2.6 cm, abaxial 0.8–1.8 vs. 2.3–2.7 cm) with confluent thecae at apex (vs. not confluent).

**Figure 1.**
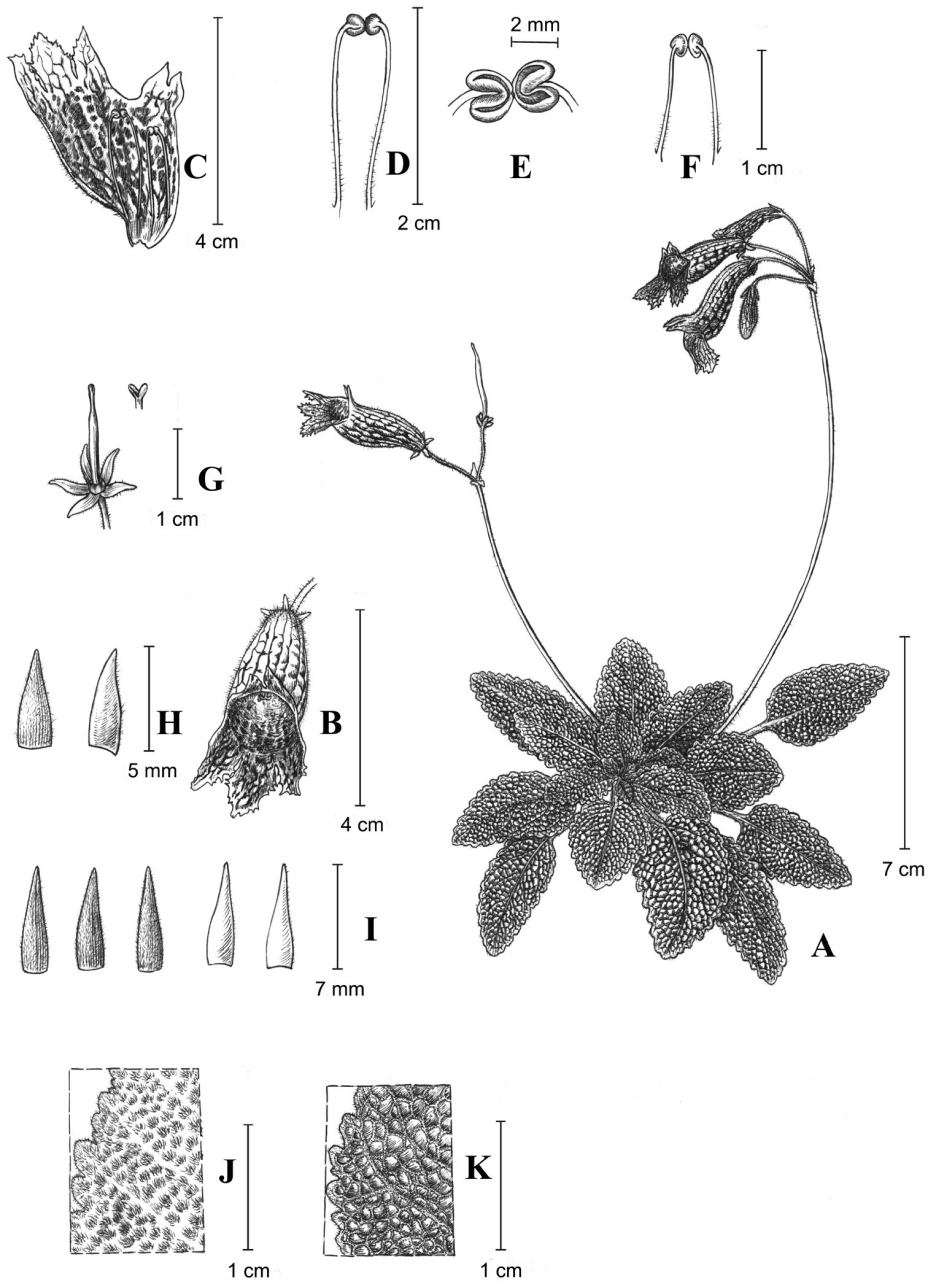
*Oreocharisodontopetala*. **A** habit; **B** flower; **C** opened corolla, showing lip lobes and stamens; **D** abaxial stamens (dorsal view); **E** cohering pair of anthers (anterior view); **F** adaxial stamens (dorsal view); **G** calyx, pistil and stigma; **H** bracts (ventral and dorsal view); **I** sepals (the two on the right showing ventral view and the three on the left showing dorsal view); **J** adaxial leaf surface; **K** abaxial leaf surface. Drawn by Ms Yun-Xiao Liu based on the holotype (*WYQ-2018-112*).

**Figure 2.**
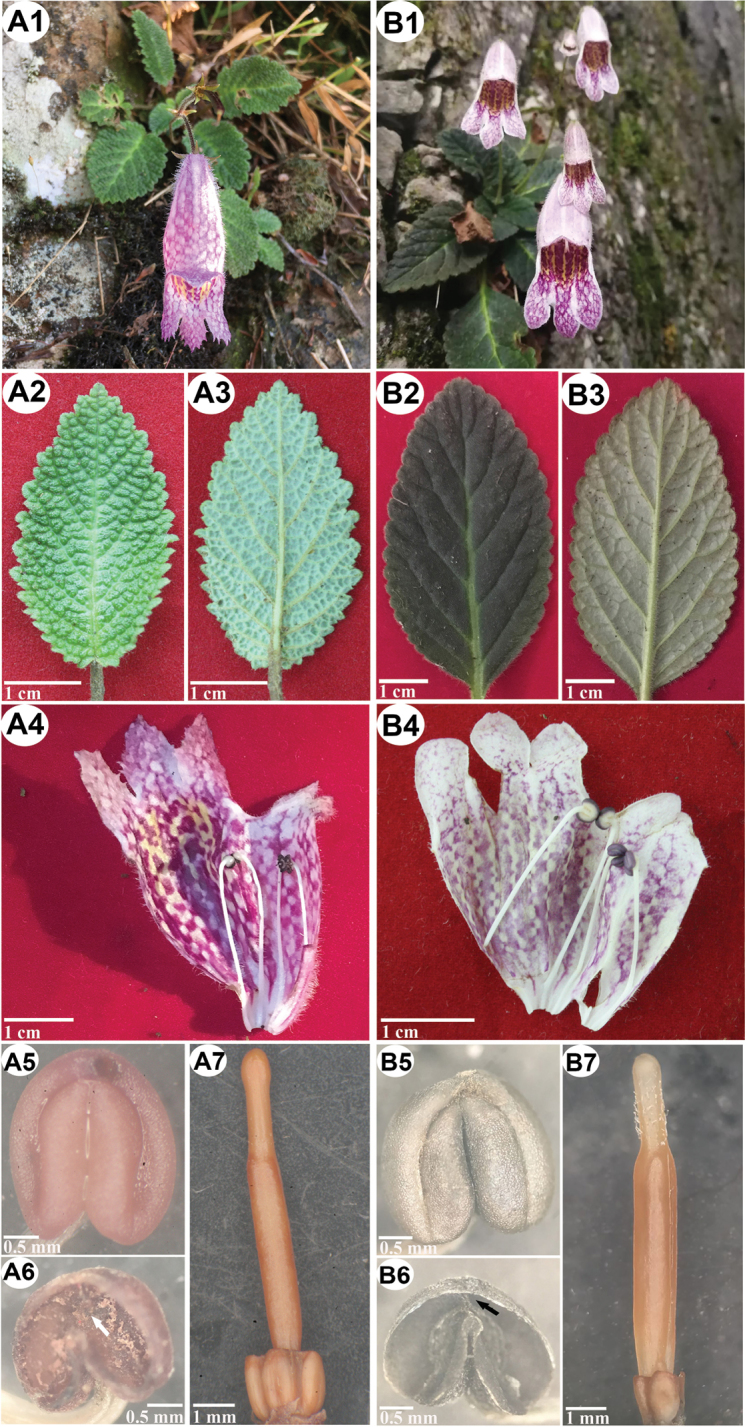
Morphological comparison of (**A**) *Oreocharisodontopetala* and (**B**) *O.elegantissima*. -**1** habitat and habit. -**2** adaxial leaf surface. -**3** abaxial leaf surface. -**4** opened corolla, showering lip lobes and stamens with anthers cohering in pairs. -**5** anthers. -**6** thecae, showing confluence at apex (white arrowhead), or no confluence (black arrowhead). -**7** immature pistil and disc, showing glandular pubescence (**B**) or absence (**A**).

#### Type.

CHINA. Guizhou Province: Panzhou City, Wumeng Town, Wumeng grassland, growing on limestone rocks in hills, 26°4.33'N, 104°37.34'E, alt. 2100 m, 14 August 2018, *Ying-Qiang Wang*, *WYQ-2018-112* (holotype: SN!; isotypes: SN!).

#### Description.

*Perennial herbs*, rosette forming. *Rhizomes* straight, terete, 0.8–2.1 cm long, ca. 0.8–1.1 cm in diameter. *Leaves* 8–18, basal; *leaf blade* papyraceous, usually ovate, rarely narrowly ovate and broadly ovate, 2.4–6.4(–7.2) × 1.3–3.5(–4.0) cm, apex acute, base rounded to shallowly cordate, margin crenate-serrate, adaxially green, bullate, white pubescent except veins, abaxially pale green, rust-brown sericeous along midrib and lateral veins, white pubescent along veinlets; *lateral veins* 4–7 pairs per side, adaxially inconspicuous and slightly concave, abaxially prominent, reticulate veinlets conspicuous; *petiole* 0.4–5.4(–7.0) cm, outer leaves with long petiole, densely rust-brown sericeous. *Cymes* 1–6, axillary, 1–2-branched, 1–6(–11)-flowered, each plant bearing 1–18(–22) flowers; *peduncle* 5.0–15.3(–19.3) cm long, 0.7–1.5(–2.1) mm in diameter, brown-purple, brown villous; *bracts* 2, opposite, green, lanceolate, (2.5–)3.3–8.0 × 1.0–2.7 mm, outside densely brown villous, inside glabrous, apex acute, margin entire. *Pedicel* 1.7–3.5 cm long, ca. 0.6–0.9(–1.2) mm in diameter, brown-purple, densely white glandular pubescent. *Calyx* 5-parted to near base, segments lobes equal, lanceolate to narrowly lanceolate, 4.2–7.3 × 1.4–1.8 mm, outside brown villous, inside glabrous, apex acute, margin entire. *Corolla* purple-red to purple, outside densely white glandular pubescent and sparsely villous, inside densely white glandular pubescent, 2.2–4.8 cm long; *tube* narrowly campanulate, gibbous abaxially, inside yellow and purple-red spotted, 1.4–2.9 cm long, 0.7–1.2 cm in diameter at middle; *limb* 5-lobed, zygomorphic, distinctly 2-lipped, adaxial lip 4.8–8.2 mm, nearly erect, 2-lobed to nearly middle, lobes triangular, apex acute, 2.0–4.1 × 2.0–3.8 mm, abaxial lip (0.8–)1.1–1.9 cm, 3-lobed to middle, lobes elliptic to ovate, margin with 4–10 long teeth, central lobe 5.6–9.8 × 3.2–7.6 mm, lateral lobes 4.0–8.6 × 3.1–6.3 mm. *Stamens* 4, coherent in pairs, included, adaxial stamens 0.5–1.4 cm, adnate to 4.5–8.3 mm above corolla base, abaxial stamens 0.8–1.8 cm, adnate to 3.5–7.0 mm above corolla base; *filaments* linear, slender, white glandular pubescent; *anthers* reniform, basifixed, glabrous, 1.9–3.1 × 1.8–3.0 mm, thecae 2, parallel, confluent at apex; *staminode* 1, 1.1–1.8 mm long, adnate to 2.7–4.6 mm above corolla base. *Disc* stellate ring-like, yellow-green, 1.5–2.5 mm high. *Pistil* 0.7–1.2 cm long at flower opening and 1.5–2.5 cm long at maturity, glabrous; *ovary* linear, 0.4–0.9 cm long at flower opening and 1.1–1.9 cm long at maturity, 0.9–1.1 mm in diameter, 1-loculed, placentas 2, parietal, projecting inwards, 2-cleft; *style* 1.8–2.7 mm at flower opening and 3.3–5.4 mm long at maturity; *stigma* 2, equal, 2-lipped, undivided, lingulate, apex obtuse, 0.6–0.9 mm long at flower opening and 1.4–1.6 mm at maturity. *Capsule* linear, straight, glabrous, ca. 37.2×1.7 mm, dehiscing septicidally by two valves at maturity.

#### Distribution and habitat.

*Oreocharisodontopetala* is only known from the type locality on Wumeng grassland, Wumeng Town, Panzhou City, Guizhou Province, China, 26°4.33'–26°8.62'N, 104°37.34'–104°36.35'E, alt. ca. 2100–2400 m.

#### Ecology and phenology.

The plants grow on limestone rocks of a hillside forest. Flowering in early August to late September, fruit ripe during early-September to October.

#### Conservation status.

Based on our field investigations, the new species is currently only known from the type locality Wumeng grassland. Only ca. 300 mature individuals were present and the extent of occurrence is estimated to be ca. 5000 m^2^. The location is not in a protected area and is accessible to casual hikers. According to the guidelines for using the IUCN Red List Categories and Criteria (IUCN 2017), the species is categorised as Endangered [EN B1abc(iv); C2a(i,ii)] due to its rarity and the threat of disturbance.

#### Etymology.

The species is named after its abaxial strongly toothed corolla lobes.

#### Vernacular name.

Chǐ Bàn Cū Tǒng Jǜ Tái (Chinese pronunciation); 齿瓣粗筒苣苔 (Chinese name).

#### Morphology (SEM) of leaf epidermis and epidermal cells of style

**Figure 3.**
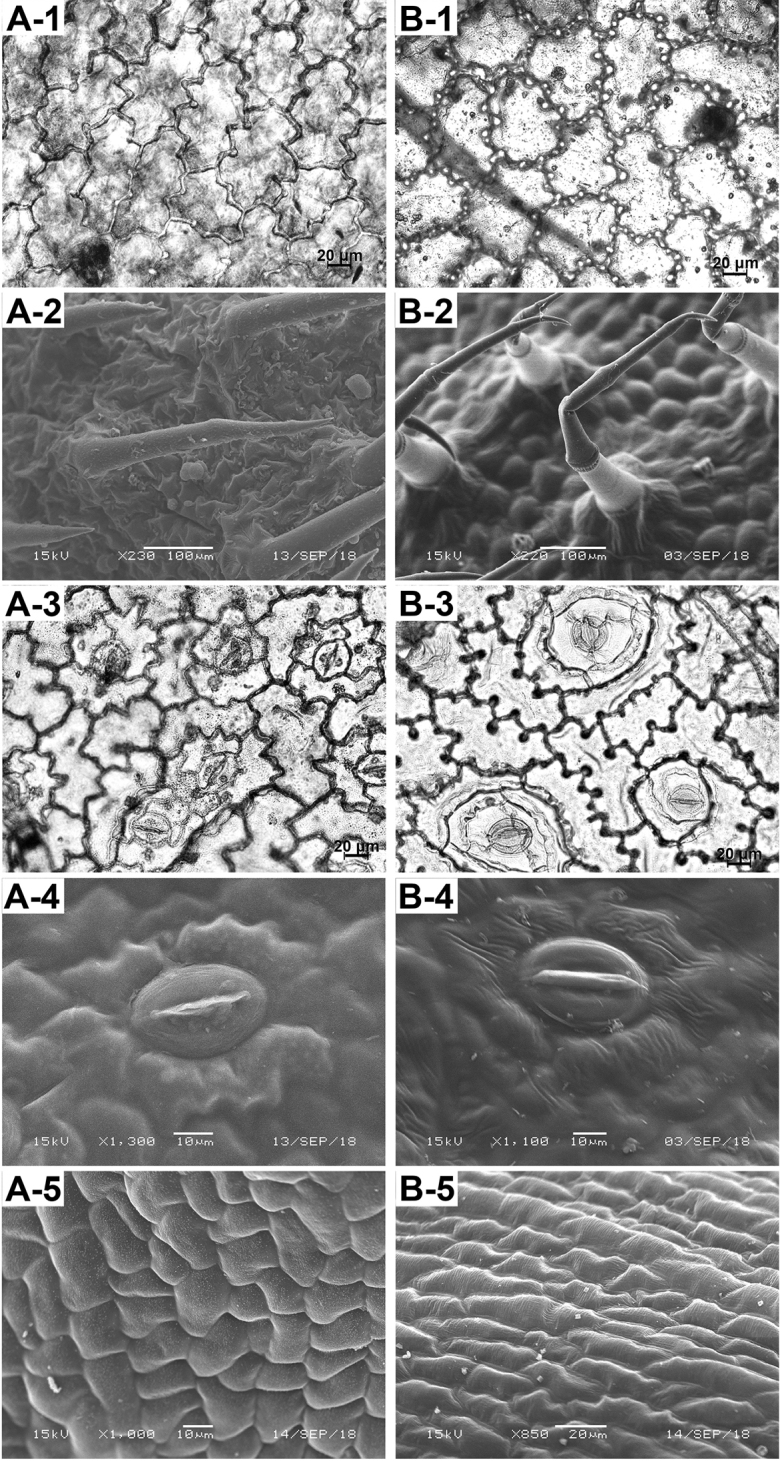
Comparative morphology (LM & SEM) of leaf epidermal surface and epidermal cells of style between (**A**) *Oreocharisodontopetala* and (**B**) *O.elegantissima*. -**1** adaxial leaf epidermal cells (LM). -**2** adaxial leaf epidermal trichome and its cuticular membrane (SEM). -**3** abaxial leaf epidermal cells (LM). -**4** abaxial leaf cuticular membrane and stomata (SEM). -**5** style epidermal cell shape and ornamentation (SEM).

(Fig. 3). The leaf epidermal cells of *Oreocharisodontopetala* on both adaxial and abaxial sides were irregular, with smooth cuticular membranes and sinuate anticlinal walls (Fig. 3A1–4). The epidermal trichomes on both adaxial and abaxial leaf blades were multicellular, with rugulate membranes (Fig. 3A2). The stomata apparata were only found on the abaxial epidermis and were assigned to the anisocytic type, with rugulate membranes, stomatal length 34.8 ± 3.4 (29.3–43.6) μm, stomatal width 26.9 ± 2.8 (21.7–31.7) μm (Fig. 3A4). The outer stomatal rims were striate (Fig. 3A4). The epidermal cells of the style were quadrilateral or polygonal, with striate cuticular membranes and many granular derivatives (Fig. 3A5).

#### Notes.

It is worth noting that *Oreocharisodontopetala* shares the narrowly campanulate, abaxial gibbose corolla tube, anthers coherent in pairs at apex, as well as similar ovary structure with all other species of the former *Briggsia*. It is most similar to *O.elegantissima*, but is distinct from its congeners by its adaxially bullate leaf blade, abaxially conspicuous reticulate veinlets, brown-purple peduncles, triangular adaxial corolla lobes and abaxial corolla lobe margins bearing 4–10 long teeth, glabrous style and shorter stamens with confluent thecae at the apex, as well as the leaf epidermal characters. The detailed morphological comparison between *O.odontopetala* and *O.elegantissima* is provided in Tables 1, 2.

Furthermore, the characteristic, abaxial corolla lobe margins with 4–10 long teeth, is easily distinguished from other species of the enlarged *Oreocharis*. The bullate leaf is quite rare in the enlarged *Oreocharis* and only occurs in the new species and other few species such as *O.bullata*, *O.curvituba*, *O.glandulosa*, *O.primuliflora*, *O.magnidens* (and to a lesser degree O.×heterandra), but it has a very different corolla, style and stamen amongst these species (Li and Wang 2004; Wei et al. 2016). The stellate ring-like disc is also rare in the enlarged *Oreocharis* and only found in the new species, *O.elegantissima* and *O.duyunensis* (Guo et al. 2018). Therefore, this new species has a unique morphology amongst the species of the extended *Oreocharis*.

**Table 1.** Morphological comparison between *Oreocharisodontopetala* and *O.elegantissima*.

Character	* O. odontopetala *	* O. elegantissima *
Adaxial leaf blade	bullate	not bullate
Abaxial leaf blade	reticulate veinlets conspicuous	veinlets inconspicuous
Peduncle	brown-purple	green
Corolla	purple-red to purple with white spots on the face	white, purple with purple or white striate on the face
Corolla adaxial lip	lobes triangular, apex acute	lobes oblong, apex rounded
Corolla abaxial lip	lobe margin with 4–10 long teeth, apex acute	lobe margin nearly entire, apex rounded
Stamens	adaxial 0.5–1.4 cm long, abaxial 0.8–1.8 cm long	adaxial 2.0–2.6 cm long, abaxial 2.3–2.7 cm long
Anthers	thecae confluent at apex	thecae not confluent
Style	glabrous	glandular pubescent

**Table 2.** Morphological comparisons (SEM & LM) of leaf epidermal surface and epidermal cells of style between *Oreocharisodontopetala* and *O.elegantissima*.

Characters	* O. odontopetala *	* O. elegantissima *
Leaf epidermal cell	adaxial and abaxial cuticular membranes smooth, anticlinal walls sinuate without knobs	adaxial and abaxial cuticular membranes striate, anticlinal walls sinuate with knobs
Leaf epidermal trichome	both adaxial and abaxial membranes rugulate	adaxial membranes smooth, abaxial membranes rugulate
Stomata	smaller, 34.8±3.4 × 26.9±2.8 μm, outer stomatal rims striate	larger, 37.7±2.9 × 31.9±3.0 μm, outer stomatal rim nearly smooth
Style epidermal cells	quadrilateral or polygonal, cuticular membranes many granular derivatives	long irregulate, cuticular membranes without granular derivative

## Supplementary Material

XML Treatment for Oreocharis odontopetala
